# Calcaneal osteotomy due to insertional calcaneal tendinopathy: preoperative planning

**DOI:** 10.1186/s13018-022-03359-z

**Published:** 2022-11-05

**Authors:** Matej Mazura, Tomas Goldman, Popelka Stanislav, David Kachlik, Rastislav Hromadka

**Affiliations:** 1 First Department of Orthopaedics, First Faculty of Medicine, Charles University and Motol University Hospital, V Úvalu 84, 150 06 Prague 5, Czech Republic; 2 Department of Anatomy, Second Faculty of Medicine, Charles University, V Úvalu 84, 150 06 Prague 5, Czech Republic; 3 Department of Mechanics, Bioemchanics and Mechatronics, Faculty of Mechanical Engineering, Czech Technical University in Prague, Technická 4, 166 07 Prague 6, Czech Republic

**Keywords:** Insertional Achilles tendinopathy, Calcaneal osteotomy, Chauveaus-Liet angle, Plantar aponeurosis

## Abstract

**Purpose:**

Dorsal closing wedge calcaneal osteotomy (DCWCO) is indicated in patients with insertional tendinopathy of the calcaneal (Achilles) tendon. The Chauveaus-Liet’s (CL) angle is represented by the difference between the angle of verticalization (*α*) and morphological angle (*β*) of the calcaneus (CL angle = *α* − *β*). The purpose of the study was to assess whether the DCWCO affects the Chauveaus-Liet’s angle.

**Methods:**

The study included 12 patients indicated to DCWCO. Three directions of close wedge osteotomy were designed for each patient—horizontal, vertical and in the middle type of osteotomy and a virtual osteotomy was created in each of them in the ABAQUS system in cooperation with Czech Technical University. The most used directions of osteotomy according to the available literature were used. We evaluated *α* and *β* angles before and after osteotomy, changes of the length plantar aponeurosis and the elevation of distal insertional point of the calcaneal tendon. The changes of grades, median and standard deviation were observed.

**Results:**

The change of the alfa angle was dependent on the direction of the osteotomy and the change of the beta angle was affected by the size of the osteotomy. The greatest elevation of the distal insertional point of the calcaneal tendon occurred in the horizontal type of the osteotomy.

**Conclusion:**

Our study shows that the more we want to reduce the tension in the calcaneal tendon, the more we have to perform an osteotomy horizontally. This study could serve as a preoperative guide for osteotomy planning.

## Introduction

The insertional tendinopathy of the calcaneal (Achilles) tendon is a common cause of heel pain. The pain on the posterior part of the calcaneus can be a part of Haglund's syndrome. It usually includes retrocalcaneal bursitis and posterosuperior bony prominence of the calcaneus. The syndrome was first described in 1928 by a Swedish surgeon, Patrick Haglund.

The first-line treatment of the insertional tendinopathy is a conservative management based on physiotherapy, extracorporeal shockwave treatment, foot-wear modification, and platelet-rich plasma therapy [1, 2]. Operative treatment, including resection of the bony prominence or dorsal closing wedge calcaneal osteotomy (DCWCO) can be indicated after unsuccessful conservative treatment [3, 4]. This type of the osteotomy was published by Zadek in 1939 [5], later modified by Taylor in 1986 [6] and recently reported in the literature utilizing both open and minimally invasive techniques [7–9]. A minimally invasive calcaneal osteotomy (MICO) technique can be performed through a small stab incision measuring approximately 5 mm, which has the potential advantages of minimizing soft-tissue dissection [10]. The DCWCO changes the anatomical length of the calcaneus and elevates the distal insertional point of the calcaneal tendon to induce a mechanical advantage, consequently alleviating pain and permitting a fast recovery [11–13]. The osteotomy is increasingly used because it does not affect the insertion of the tendon.

**Fig. 1:**
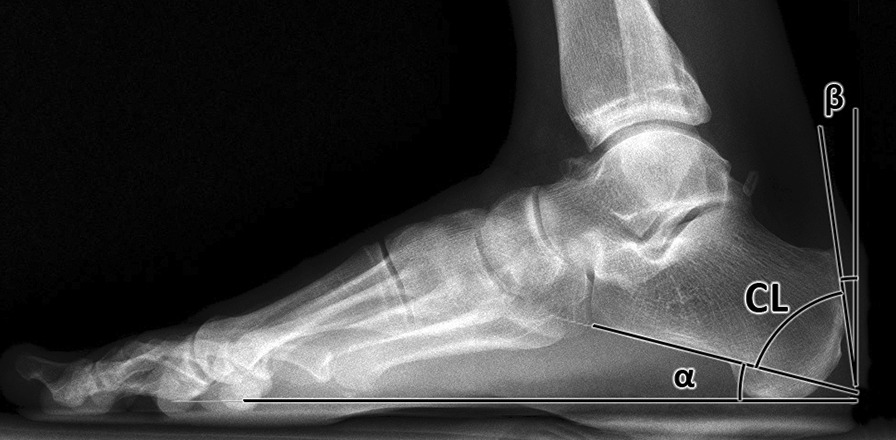
The Chauveaus-Liet's (CL) angle is represented by the difference between the angle of verticalization (*α*) and the morphological angle (*β*) of the calcaneus (CL angle = *α* − *β*)

The shape of the calcaneus may be the essence of enthesopathy. Several standard radiographic measurements have been proposed to quantify bony anomalies of the calcaneus [14–16]. The Chauveaus-Liet's angle (CL angle) is represented by the difference between the angle of verticalization (*α*) and the morphological angle (*β*) of the calcaneus (CL angle = *α* − *β*). Angle *α* is the calcaneal pitch angle or angle of verticalization of the calcaneus described as the intersection of the baseline tangent with the horizontal surface [17]. The angle *β* is formed between the vertical line tangent and the straight line joining this point to the apex of the posterosuperior crest (Fig. 1). A CL angle of more than 12° is considered abnormal, typical of the insertional tendinopathy. It is supposed that a change of the CL angle could cause different tension of the triceps surae muscle.

The X-rays of patients were used in the study for virtual preoperative measurements of the dorsal closing wedge calcaneal osteotomy. The hypothesis is that a horizontal type of the osteotomy changes notably the *α* angle and a vertical type of the osteotomy the *β* angle. The secondary objective is the magnitudes of the change of these angles in different types of osteotomies.

## Patients and methods

The study included 12 patients indicated to the DCWCO for insertional tendinopathy at the Department of Orthopaedics, First Faculty of Medicine, University Hospital Motol, Prague, Czechia. A weight-bearing X-ray of the ankle (a lateral view) was used for the preoperative planning (Fig. 1). We evaluated six scans of women and six scans of men. The average age of male patients was 45.8 years (SD ± 9.8), the average age of female patients was 49.8 (SD ± 7.0). Inclusion criteria were clinical signs of the insertional tendinopathy or retrocalcaneal bursitis, a visible “pump bump” and skin irritation by shoe-wear. All patients had failed routine conservative management for at least six months. X-ray inclusion criteria were a prominent posterior calcaneal tuberosity, an increased inclination of the calcaneus, cavus foot and degenerative changes around calcaneal tendon attachment. Exclusion criteria were as follows: history of hindfoot trauma or surgery, neuromuscular pathology (e.g. Charcot's osteoarthropathy), rheumatoid arthritis, history of calcaneal tendon trauma or surgery, posterior impingement.

**Fig. 2:**
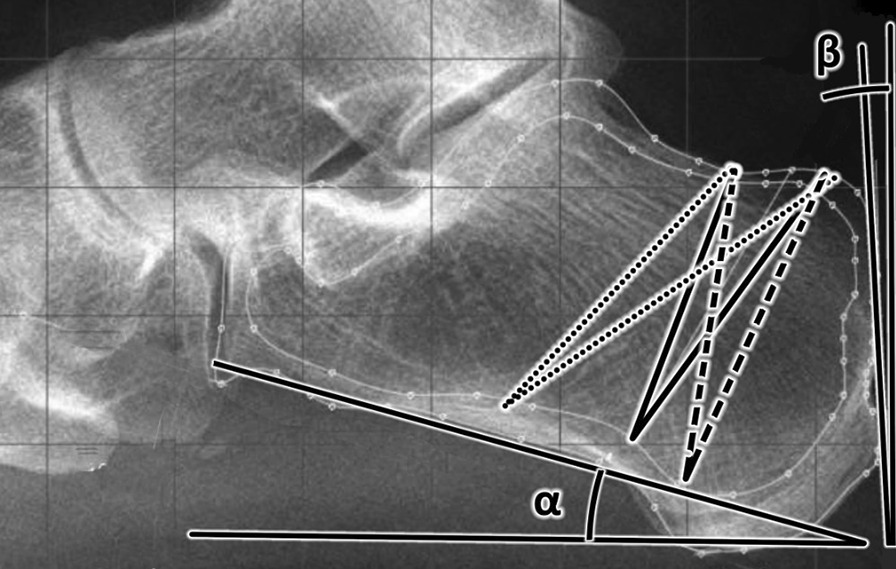
Three directions of the close wedge osteotomy which were designed for each patient—horizontal type (dotted), vertical type (dashed), and osteotomy in the middle (solid)

All X-rays were evaluated and measured in software ABAQUS/CAE (version 2019.HF9 Dassault SystemesSimulia Corp., Johnston, RI, USA) for assessing the change of the angles of interest and tension of the bone after a virtual osteotomy. Three directions of the close wedge osteotomy were designed for each patient (Fig. 2): (1) the horizontal type of the osteotomy (dotted); (2) the vertical type of the osteotomy (dashed); and (3) an osteotomy in the middle (solid). The types of osteotomies were proposed according to the current literature [4, 18, 19]. All three osteotomies were measured for the angle of 10° and 15°. The apex of each osteotomy was 5 mm above the lower cortical bone of the calcaneus.

**Fig. 3:**
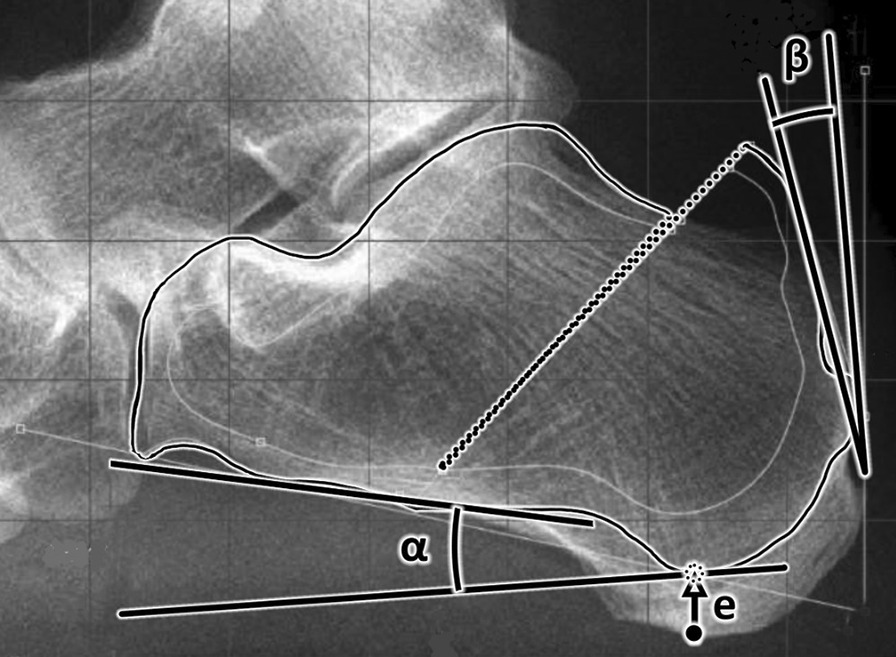
Graphs of the *α* and *β* angles changes after the osteotomy. e—elevation of the heel bone after the osteotomy

We evaluated the *α* and *β* angles on weight-bearing X-rays of patients before and after one of the proposed osteotomy (Fig. 3). On each bone the outer and inner surfaces of the cortical bone were designated for measurement of the internal tension of the calcaneus before and after the osteotomy. The distance between the medial sesamoid bone of the first metatarsophalangeal joint and the lowest part of the calcaneal tubercle was measured before and after each osteotomy as well as elevation of attachment of the calcaneal tendon.

## Results

**Fig. 4:**
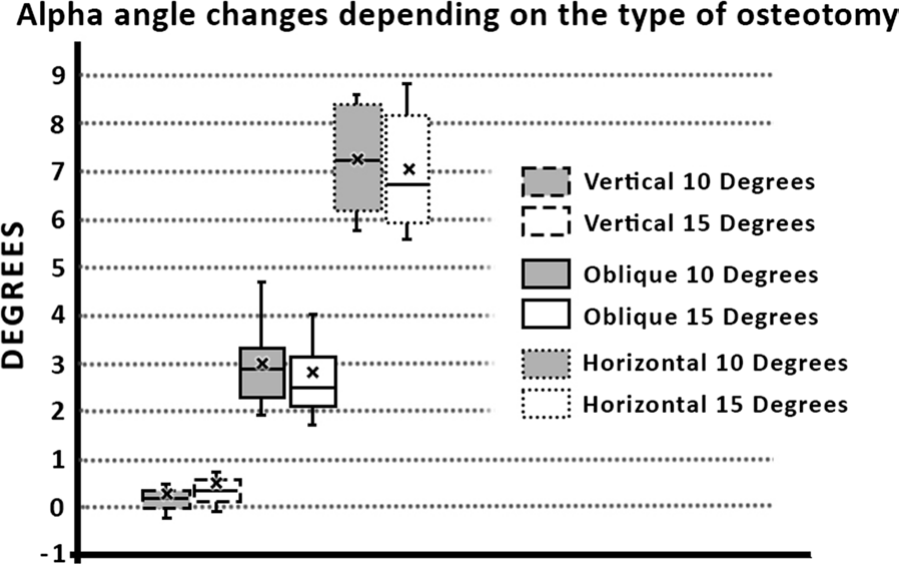
Graph of the *α* angle changes after the osteotomy

**Fig. 5:**
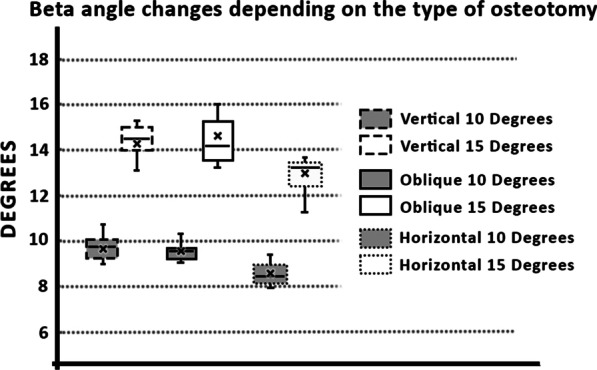
Graphs of the *β* angle changes after the osteotomy

In our study the average of the *α* angle was 22.5° (median 21.3 ± 6.5°; range from 34.5° to 9°) and the average of the *β* angle was 6.5° (median 4.4 ± 8°; range from 25.5° to − 2.4°) before the osteotomy. The average Chauveaus-Liet’s angle was 16° (median 17.6 ± 12.8°; range from 35.1° to − 9.2°). Therefore, we can state that these were abnormal values corresponding to the insertional tendinopathy. In the graphs (Figs. 4 and 5) we can clearly see the changes of the *α* and *β* angles, respectively, after osteotomies depending on different directions and size of the osteotomy.

The exact values of the angle changes are summarized in Table 1. Furthermore, changes in the length of the plantar aponeurosis are presented depending on the direction and size of the osteotomy in Table 2. This table shows the results of the elevation of the distal insertional point of the calcaneal tendon depending on the direction and size of osteotomies.

**Table 1:** Changes of alfa and beta angle after osteotomy of calcaneus

	Vertical type		Oblique type		Horizontal type	
	10°	15°	10°	15°	10°	15°
Alfa	0.4° ± 0.6°	0.6° ± 0.7°	3.0° ± 0.8°	2.8° ± 1.1°	7.2° ± 1.1°	7.0° ± 1.2°
	(1.7° to − 0.2°)	(2.6° to − 0.1°)	(4.7° to 1.9°)	(5.6° to 1.6°)	(8.6° to 5.7°)	(8.8° to 5.6°)
Beta	9.6° ± 0.9°	14.4° ± 0.7°	9.5° ± 0.4°	14.5° ± 1.3°	8.6° ± 0.5°	13.0° ± 0.8°
	(10.8° to 7.2°)	(15.3° to 13.0°)	(10.2° to 9.0°)	(17.6° to 13.1°)	(9.4° to 8.0°)	(13.7° to 11.2°)

Mean ± SD (Max to Min)

The exact values of angle changes after osteotomy. The change in the alpha angle depends on the direction of the osteotomy and the change in the beta angle is dependent on the size of the osteotomy

**Table 2:** Changes of lenght of plantar aponeurosis and elevation of calcaneus after osteotomy (mm)

	Vertical type		Oblique type		Horizontal type	
	10°	15°	10°	15°	10°	15°
AP length	− 1.1 ± 1.4	− 1.2 ± 1.7	− 0.3 ± 1.2	0.0 ± 1.9	0.4 ± 1.5	0.8 ± 2.0
	(0.0 to − 4.5)	(0.0 to − 4.9)	(1.7 to − 2.8)	(2.2 to − 3.5)	(2.5 to − 2.2)	(3.7 to − 2.8)
Elevation	0.4 ± 0.4	0.6 ± 0.6	1.8 ± 0.6	2.7 ± 0.9	4.5 ± 0.7	6.7 ± 1.1
	(1.1 to 0.0)	(1.7 to 0.0)	(2.8 to 0.9)	(4.6 to 1.4)	(5.4 to 3.0)	(8.2 to 4.7)

AP length—distance between medial sesamoid bone of first metatarsophalangeal joint and tuberosity of calcaneus; Elevation—Elevation of distal insertional point of calcaneal tendon

Mean ± SD (Max to Min)

The exact values of the change of the plantar aponeurosis length and elevation of the distal insertional point of the calcaneal tendon. A negative value of the plantar aponeurosis changes indicates a shortening. A positive value of elevation of the distal insertional point of the calcaneal tendon indicates functional relief

All proposed directions of osteotomies are reliable, and osteotomies do not change the ability to exercise.

## Discussion

The DCWCO changes the anatomical length of the calcaneus, and it elevates the distal insertional point of the calcaneal tendon to induce a mechanical advantage, consequently alleviating pain and permitting a fast recovery [11–13].The osteotomy must be fixed by one screw leading from the tuber calcanei and the axis of the screw should be perpendicular to the osteotomy and directed towards the middle of dorsal part articulatio subtalaris.

Our data demonstrated that a successful change of the CL angle, which is represented by the difference between the angle of verticalization (*α*) and the morphological angle (*β*) of the calcaneus (CL angle = *α* − *β*), could be achieved by different direction and a different size of the osteotomy direction in minimally invasive-dorsal closing wedge calcaneal osteotomy (MIS-DCWCO) (Table 1). We proved that the change of the alpha angle depends on the osteotomy direction and the alpha angle mostly affects the horizontal type of the osteotomy (dotted) (Fig. 2). On the other hand, the direction of the osteotomy does not affect the beta angle. We further showed that the size of the osteotomy affects the beta angle but does not affect the alpha angle. We could use this fact in preoperative planning, where we would consider the postoperative change depending on the morphology of the calcaneus.

The osteotomy changes the length of the plantar aponeurosis. The study measured the changing of the distance between the medial sesamoid bone of the first metatarsophalangeal joint and the lowest point of the calcaneus (Table 2). We proved that the vertical osteotomy (dashed one) has the greatest reduction in the plantar aponeurosis length. On the other hand, in the horizontal type of the osteotomy (dotted one) there has been a relative prolongation of the plantar aponeurosis. It is important to always assess the functional complex of the triceps surae muscle and plantar aponeurosis. If there is a large change in the length of the plantar aponeurosis, pain can occur in the area of the sole, known as plantar fasciitis.

The most significant elevation of the distal insertional point of the calcaneal tendon occurred in the horizontal (dotted one) type of the osteotomy. If we take into account the change in complex of the plantar aponeurosis and triceps surae muscle, the horizontal type of the osteotomy seems to be the most advantageous for DCWCO.

Insertional tendinopathy is caused by chemical attrition and bony mechanical abrasion [4]. The first-line treatment of the insertional tendinopathy is a conservative management. Operative treatment is indicated after six months of unsuccessful conservative treatment.

There are several operative treatments for refractory insertional tendinopathy. Surgical treatment can be divided into open and mini-invasive procedures. Open resection of the Haglund's deformity is still a standard procedure [20]. However, open procedures are associated with a high rate of complications. [4, 21]. The second group of surgical treatment consists in a minimally invasive procedure. The first option is the endoscopic bony and soft-tissue decompression with arthroscopic shaver [22, 23]. The second mini-invasive surgical treatment option is the minimally invasive-dorsal closing wedge calcaneal osteotomy (MIS-DCWCO), which we focused on in our study. We proved that certain types of the osteotomy affect the change of alfa and beta angles which should reduce pain.

## Conclusion

The principle of close wedge osteotomies is to change the anatomical length of the calcaneus. It elevates the distal insertional point of the calcaneal tendon to induce a mechanical advantage, consequently alleviating pain and permitting a fast recovery what is known from the previous studies [11–13]. But no previous research has published the most usable direction and the apex of the osteotomy. We proved that the change of the alpha angle depends on the osteotomy direction. Our study results show that the more we want to reduce the tension in the calcaneal tendon, the more horizontally we have to perform the osteotomy. We confirmed this statement on an anatomical model by performing virtual osteotomies. This study could serve as a preoperative guide for osteotomy planning for patients with insertional tendinopathy.

### Study limitations

The present study has several limitations, such as the relatively small cohort and absence of clinical symptoms. Despite these limitations, this study serves as an anatomically based operational planning aid for patients with insertional tendinopathy and input data for a large clinical study.

## Data Availability

All data are available from the author.

## Abbreviations

CL: Chauveaus-Liet’s angle
OT: Osteotomy
DCWCO: Dorsal closing wedge calcaneal osteotomy
MICO: Minimally invasive calcaneal osteotomy
MIS-DCWCO: Minimally invasive-dorsal closing wedge calcaneal osteotomy

## References

[1] Erroi D, Sigona M, Suarez T, Trischitta D, Pavan A, Vulpiani MC, Vetrano M (2017). Conservative treatment for Insertional Achilles Tendinopathy: platelet-rich plasma and focused shock waves. A retrospective study. Muscles Ligaments Tendons J.

[2] Notarnicola A, Maccagnano G, Tafuri S, Forcignano MI, Panella A, Moret B (2014). CHELT therapy in the treatment of chronic insertional Achilles tendinopathy. Lasers Med Sci.

[3] Irwin TA (2010). Current concepts review: insertional achilles tendinopathy. Foot Ankle Int.

[4] Lui TH, Lo CY, Siu YC (2019). Minimally invasive and endoscopic treatment of haglund syndrome. Foot Ankle Clin.

[5] Schoolfield BL (1939). An operation for the cure of flatfoot. Ann Surg.

[6] Taylor GJ (1986). Prominence of the calcaneus: Is operation justified?. J Bone Jt Surg Br.

[7] Georgiannos D, Lampridis V, Vasiliadis A, Bisbinas I (2017). Treatment of insertional Achilles pathology with dorsal wedge calcaneal osteotomy in athletes. Foot Ankle Int.

[8] Maynou C, Mestdagh H, Dubois HH, Petroff E, Elise S. Is calcaneal osteotomy justified in Haglund's disease?. Rev Chir Orthop Reparatrice Appar Mot. 1998;84(8):734–738. Retrieved from https://www.ncbi.nlm.nih.gov/pubmed/10192124. 10192124

[9] Tourne Y, Baray AL, Barthelemy R, Moroney P (2018). Contribution of a new radiologic calcaneal measurement to the treatment decision tree in Haglund syndrome. Orthop Traumatol Surg Res.

[10] Jowett CR, Rodda D, Amin A, Bradshaw A, Bedi HS (2016). Minimally invasive calcaneal osteotomy: a cadaveric and clinical evaluation. Foot Ankle Surg.

[11] Boffeli TJ, Peterson MC (2012). The keck and kelly wedge calcaneal osteotomy for Haglund's deformity: a technique for reproducible results. J Foot Ankle Surg.

[12] Nordio A, Chan JJ, Guzman JZ, Hasija R, Vulcano E (2020). Percutaneous Zadek osteotomy for the treatment of insertional Achilles tendinopathy. Foot Ankle Surg.

[13] Vernois J, Redfern D, Ferraz L, Laborde J (2015). Minimally invasive surgery osteotomy of the hindfoot. Clin Podiatr Med Surg.

[14] Heneghan MA, Pavlov H. The Haglund painful heel syndrome. Experimental investigation of cause and therapeutic implications. Clin Orthop Relat Res. 1984; (187): 228–234. Retrieved from https://www.ncbi.nlm.nih.gov/pubmed/6744723. 6744723

[15] Husson JL, De Korvin B, Polard JL, Attali JY, Duvauferrier R. Study of the correlation between magnetic resonance imaging and surgery in the diagnosis of chronic Achilles tendinopathies. Acta Orthop Belg. 1994;60(4): 408–412. Retrieved from https://www.ncbi.nlm.nih.gov/pubmed/7847091. 7847091

[16] Ruch JA (1974). Haglund's disease. J Am Podiatry Assoc.

[17] DiGiovanni JE, Smith SD (1976). Normal biomechanics of the adult rearfoot: a radiographic analysis. J Am Podiatry Assoc.

[18] Ge Z, Ma L, Tang H, Yang M, Yang A, Yuan C, Chen W (2020). Comparison of dorsal closing wedge calcaneal osteotomy versus posterosuperior prominence resection for the treatment of Haglund syndrome. J Orthop Surg Res.

[19] Tourne Y, Baray AL, Barthelemy R, Karhao T, Moroney P (2021). The Zadek calcaneal osteotomy in Haglund's syndrome of the heel: clinical results and a radiographic analysis to explain its efficacy. Foot Ankle Surg.

[20] McAlister JE, Hyer CF (2015). Safety of achilles detachment and reattachment using a standard midline approach to insertional enthesophytes. J Foot Ankle Surg.

[21] Phisitkul P (2012). Endoscopic surgery of the Achilles tendon. Curr Rev Musculoskelet Med.

[22] Ortmann FW, McBryde AM (2007). Endoscopic bony and soft-tissue decompression of the retrocalcaneal space for the treatment of Haglund deformity and retrocalcaneal bursitis. Foot Ankle Int.

[23] Vega J, Baduell A, Malagelada F, Allmendinger J, Dalmau-Pastor M (2018). Endoscopic Achilles tendon augmentation with suture anchors after calcaneal exostectomy in haglund syndrome. Foot Ankle Int.

